# Supplementary material for: In silico Pathway Activation Network Decomposition Analysis (iPANDA) as a method for biomarker development
Source: Nat Commun. 2016 Nov 16;7:13427. doi: 10.1038/ncomms13427 (PMC5116087; doi:10.1038/ncomms13427)
Supplement: Supplementary Information — Supplementary Figures 1-9, Supplementary Tables 1-3, Supplementary Notes 1-2 and Supplementary References. [file ncomms13427-s1.pdf]

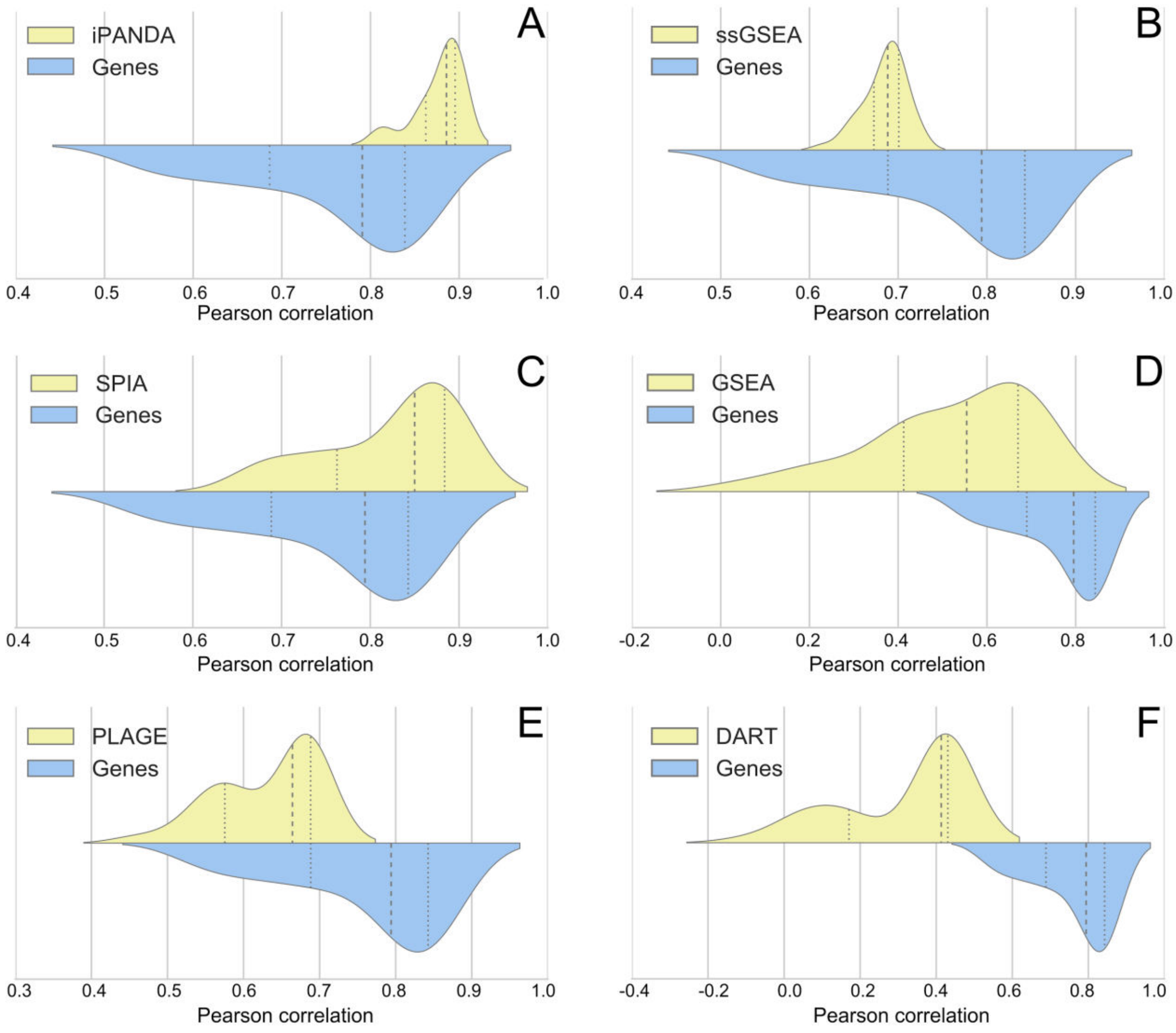

Supplementary Figure 1. **Sample-wise similarity between data obtained using various profiling platforms.** Pearson cross-correlations between gene expression levels obtained with Affymetrix and Agilent gene expression analysis platforms for the same set of samples are shown in blue. Cross-correlations between corresponding pathway activation (enrichment) values calculated using six different pathway based transcriptomic data analysis algorithms are shown in yellow. Data samples were obtained from MicroArray Quality Control (MAQC) dataset.

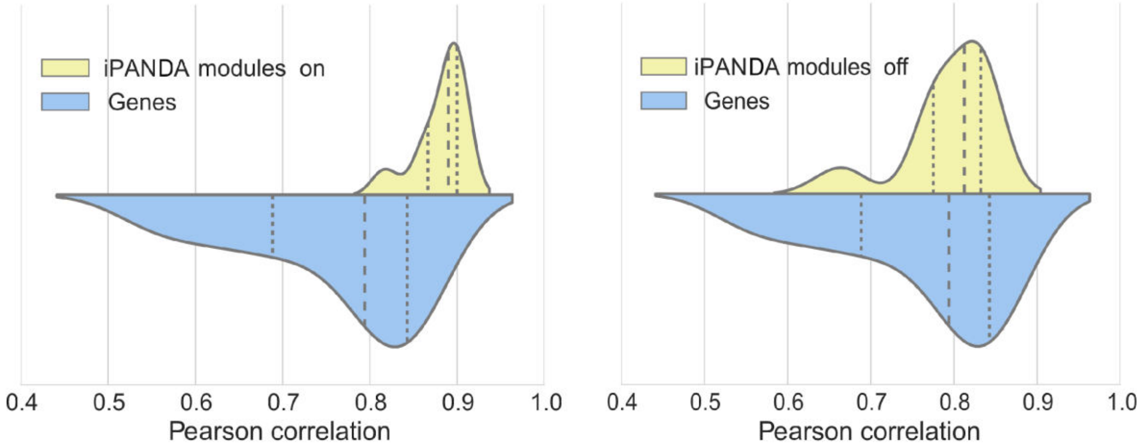

Supplementary Figure 2. Sample-wise similarity between data obtained using various profiling platforms with modules on/off. Pearson cross-correlations between gene expression levels obtained with Affymetrix and Agilent gene expression analysis platforms for the same set of samples are shown in blue. Cross-correlations between corresponding pathway activation (enrichment) values calculated using iPANDA (left) and iPANDA with feature for grouping genes into modules "switched off", meaning that all genes are considered individually and no information from COEXPRESSdb is being utilized (right), are shown in yellow. Data samples were obtained from MicroArray Quality Control (MAQC) dataset.

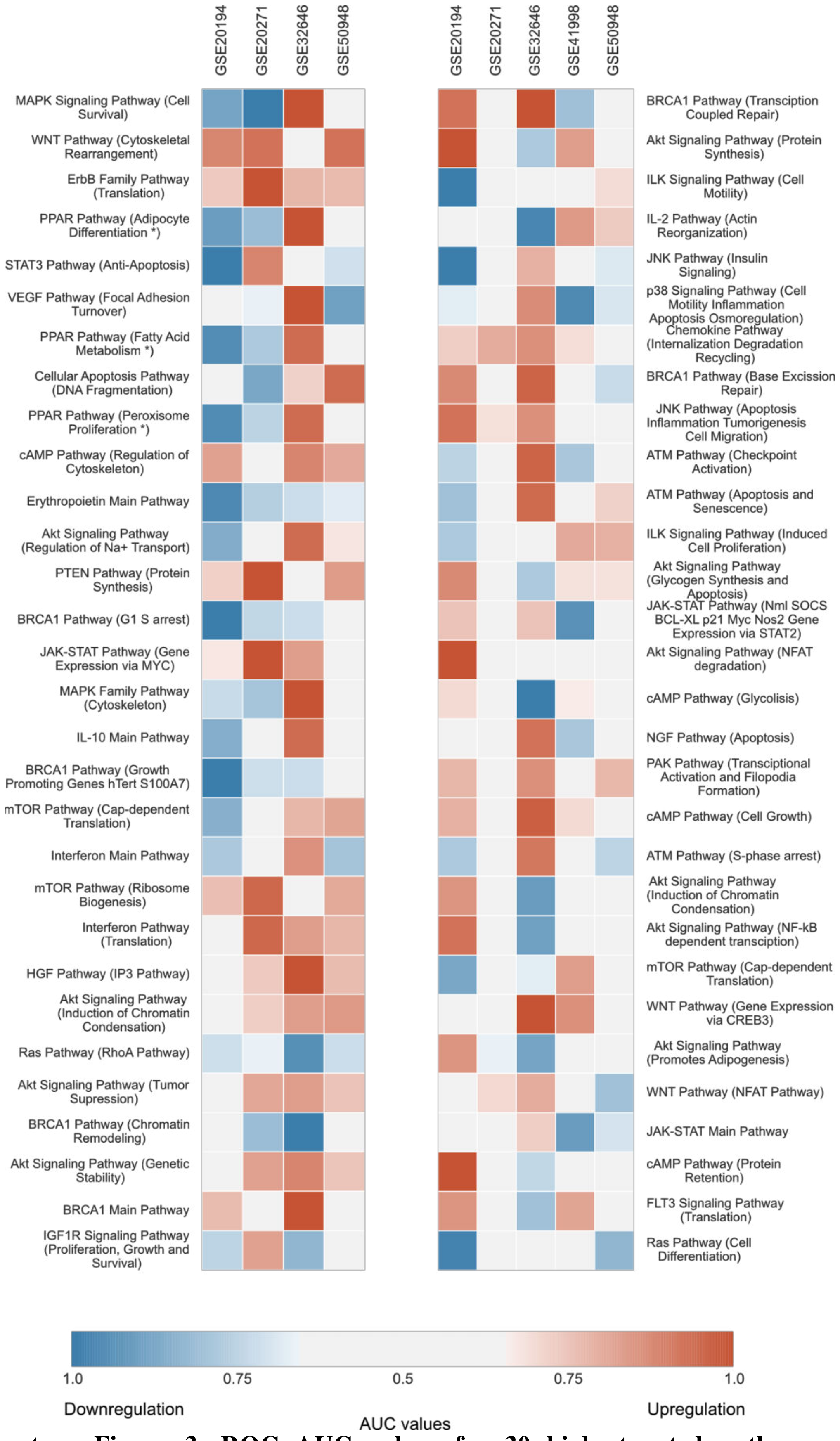

Supplementary Figure 3. **ROC AUC values for 30 highest rated pathway markers.** Pathway markers of responders/non-responders to Paclitaxel ERN HER2P (left) and ERN HER2N (right) breast cancer treatment obtained using GSEA.

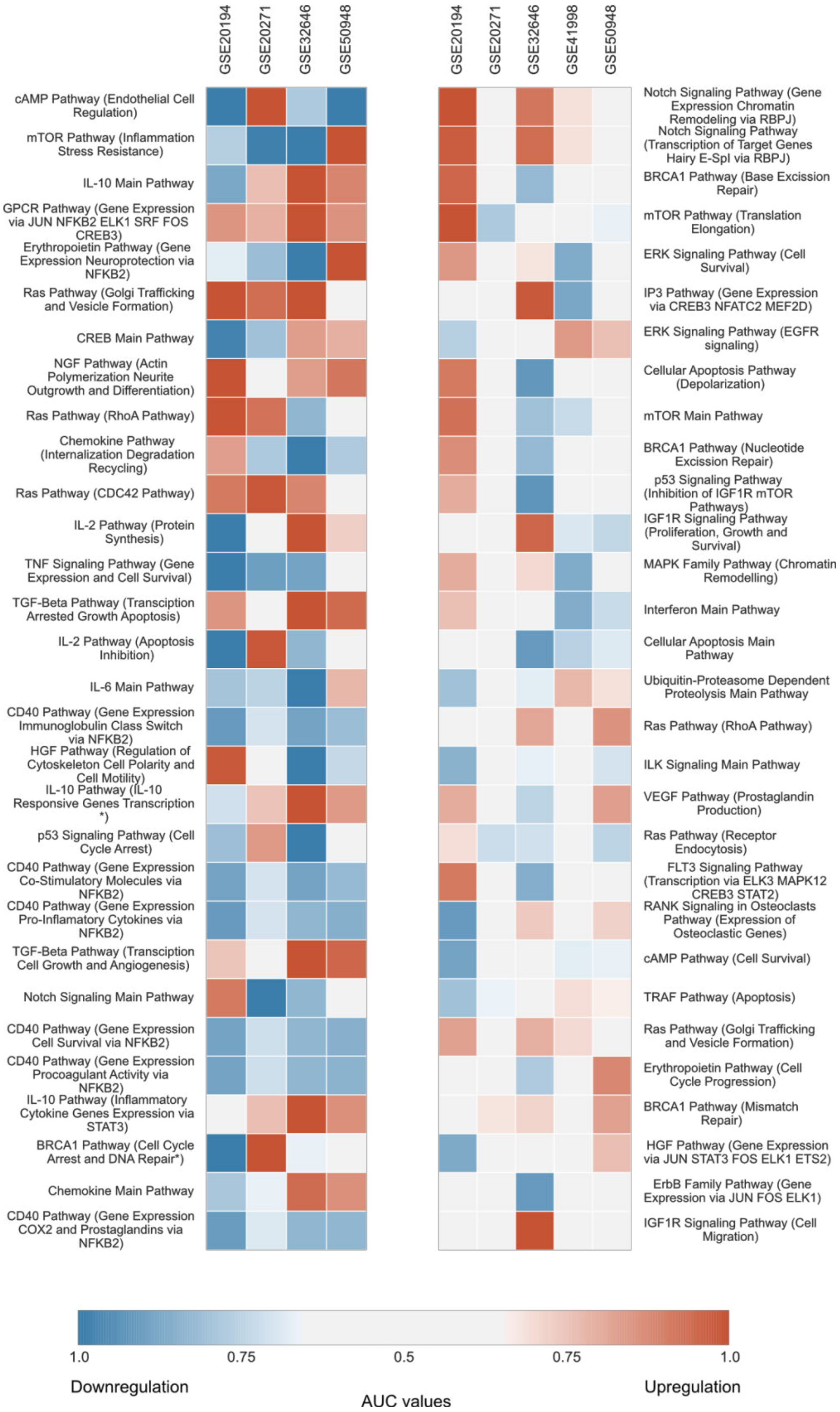

Supplementary Figure 4. **ROC AUC values for 30 highest rated pathway markers.** Pathway markers of responders/non-responders to Paclitaxel ERN HER2P (left) and ERN HER2N (right) breast cancer treatment obtained using SPIA.

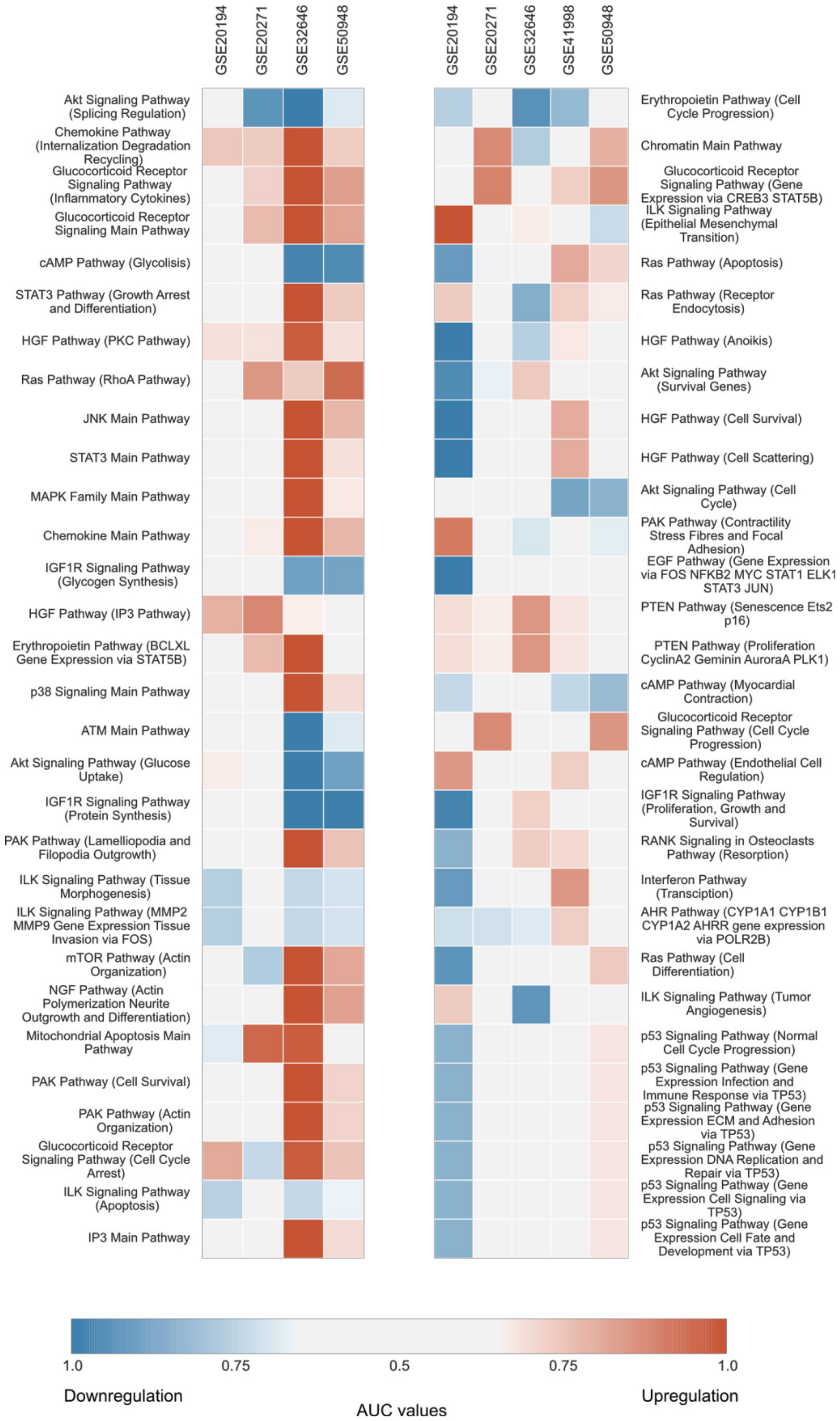

Supplementary Figure 5. **ROC AUC values for 30 highest rated pathway markers.** Pathway markers of responders/non-responders to Paclitaxel ERN HER2P (left) and ERN HER2N (right) breast cancer treatment obtained using ssGSEA.

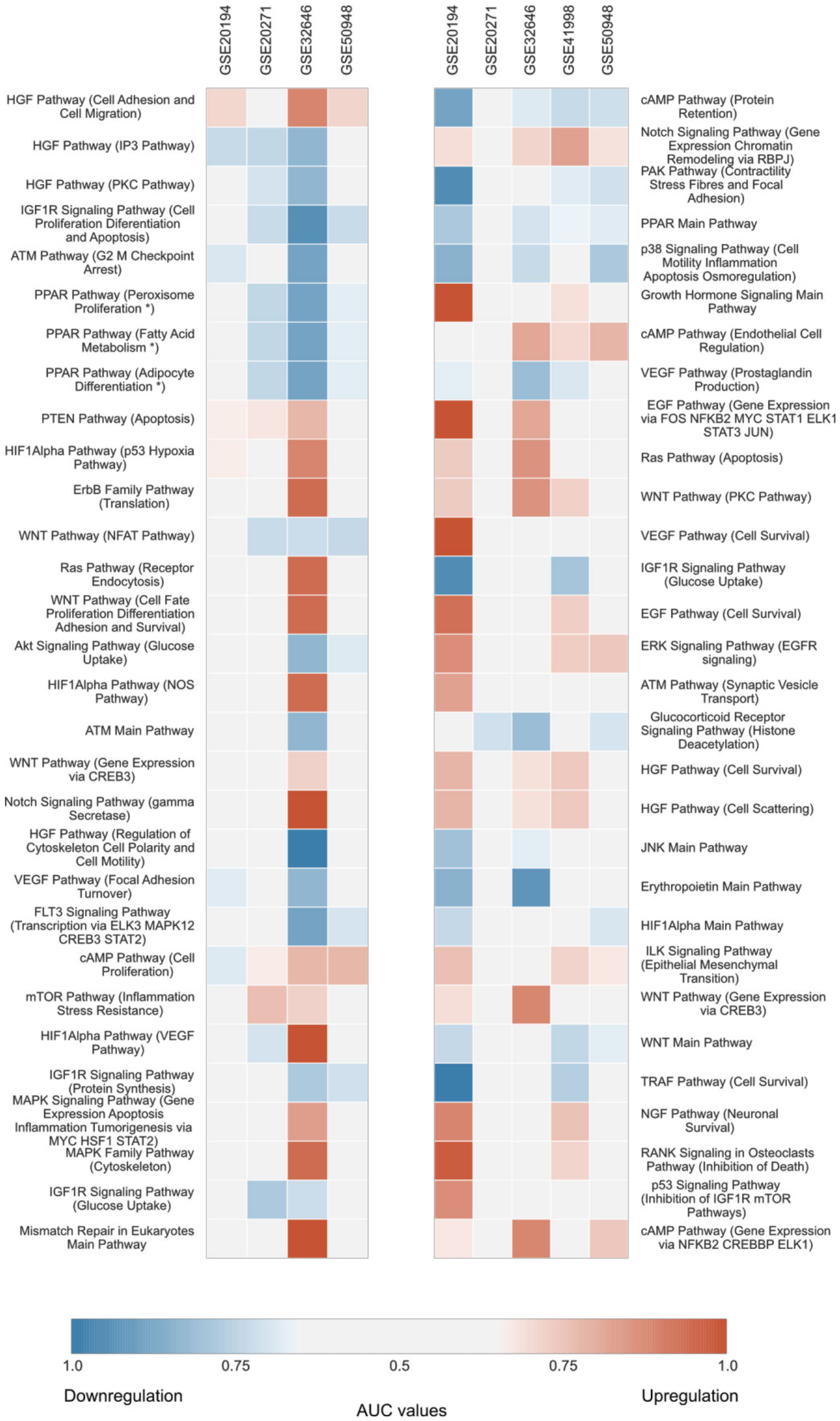

Supplementary Figure 6. **ROC AUC values for 30 highest rated pathway markers.** Pathway markers of responders/non-responders to Paclitaxel ERN HER2P (left) and ERN HER2N (right) breast cancer treatment obtained using PLAGE.

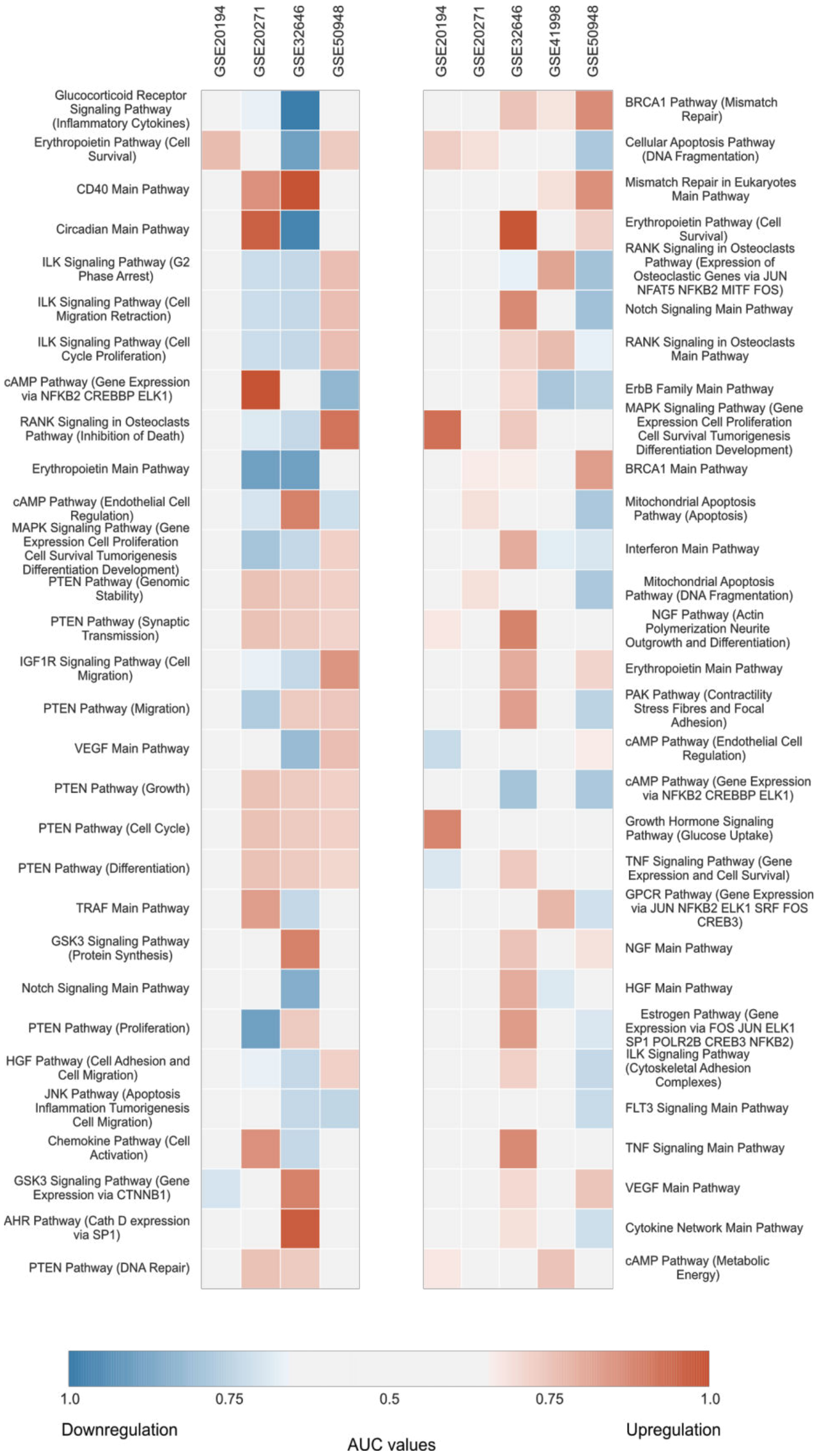

Supplementary Figure 7. **ROC AUC values for 30 highest rated pathway markers.** Pathway markers of responders/non-responders to Paclitaxel ERN HER2P (left) and ERN HER2N (right) breast cancer treatment obtained using DART.

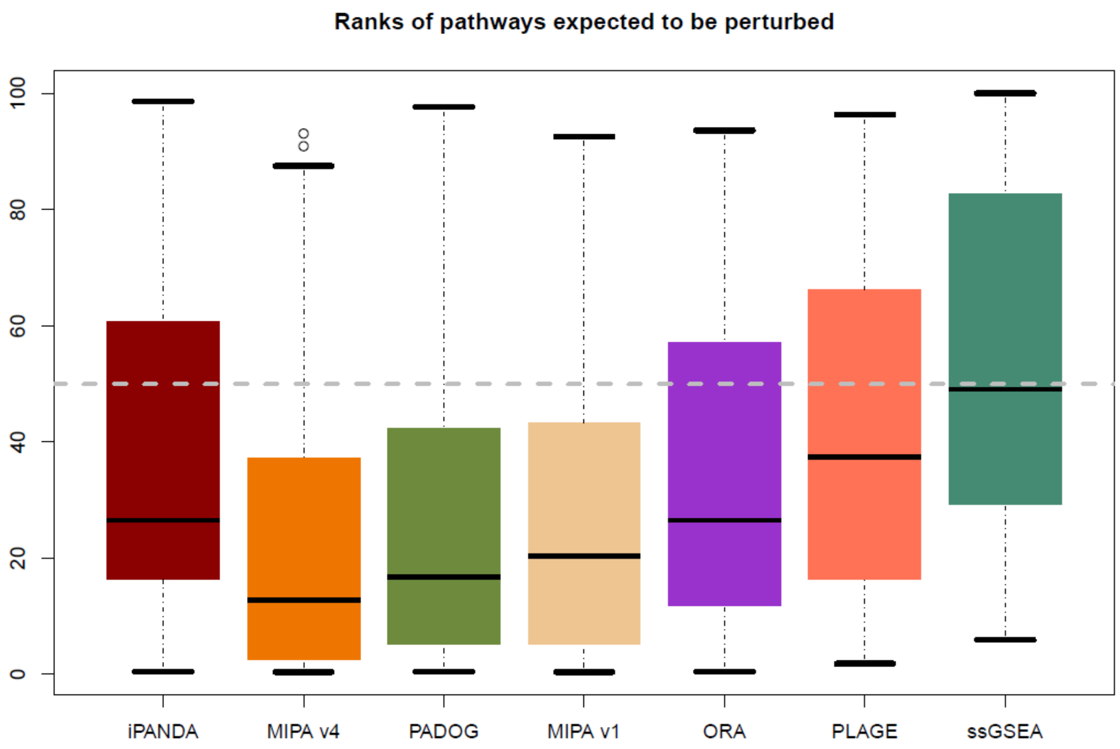

Supplementary Figure 8. Results of benchmarking according to recently proposed pipeline[1] for methods of pathway analysis in respect to prioritization criteria. Ranks of pathways expected to be perturbed in a set of transcriptomic datasets for the following pathway analysis methods: iPANDA, PADOG[2], ORA[3], PLAGE[4], ssGSEA[5] and two versions of MIPA[6] framework (v1 and v4). Relative ranks (100 * rank of the pathway / total number of pathways analyzed, where a lower rank indicates more significant perturbation of the pathway) were calculated for each method.

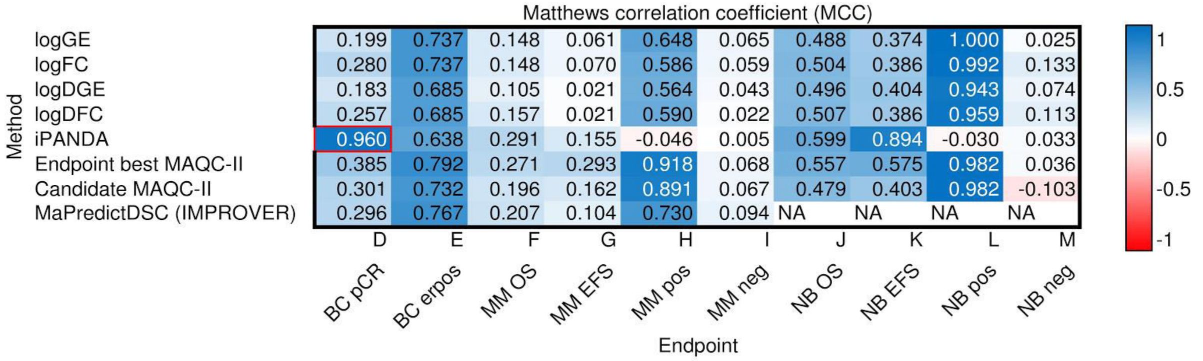

Supplementary Figure 9. **Performance of random forest based classifiers trained using sample-wise iPANDA scores and four different gene expression subsets**. Expression levels of all genes, fold change for all genes between the training set and paired normals, expression levels of most differentially expressing genes (t-test $p < 0.05$), and fold change in expression levels of most differentially expressing genes (t-test $p < 0.05$) between the training and normal tissue datasets on cancer-related MAQC-II endpoints (Endpoint names are given according to original MAQC-II study). The results are compared to performance of corresponding gene-level predictors from MAQC-II and IMPROVER teams. The result of iPANDA-based classifier for endpoint D is in a red box, because the performance of iPANDA algorithm on data used for this endpoint was utilized during iPANDA design.

Supplementary Tables

Supplementary Table 1. MAQC-II data sets and corresponding normal controls selected for calculations.

MAQC-II Endpoint	GEO Series	Normal Control Series
D	GSE20194	GSE9574
E	GSE20194	GSE9574
F	GSE24080	GSE13591
G	GSE24080	GSE13591
H	GSE24080	GSE13591
I	GSE24080	GSE13591
J	GSE49710	GSE19422
K	GSE49710	GSE19422
L	GSE49710	GSE19422
M	GSE49710	GSE19422

Supplementary Table 2. Transcriptomic data sets on paclitaxel breast cancer treatment utilized in the study.

Breast cancer tissue data sets (total number of samples)	Normal tissue data sets (number of samples used)	Affymetrix platform
GSE20194 (241)	GSE9574 (15)	GPL96
GSE20271 (44)	GSE9574 (15)	GPL96
GSE22513 (28)	GSE42568 (10)	GPL570
GSE32646 (99)	GSE42568 (10)	GPL570
GSE41998 (116)	GSE10797 (10)	GPL571
GSE50948 (56)	GSE42568 (10)	GPL570

Supplementary Table 3. The number of ERN HER2P, ERN HER2N and overall number of breast cancer samples from patients treated with paclitaxel with a distinct outcome.

GEO ID	Number of responders ERN HER2P	Number of non-responders ERN HER2P	Number of responders ERN HER2N	Number of non-responders ERN HER2N	Total number of responders	Total number of non-responders
GSE20194	15	14	27	46	49	192
GSE20271	6	3	10	25	16	28
GSE22513	N/A	N/A	N/A	N/A	8	20
GSE32646	9	9	10	16	24	75
GSE41998	0	0	28	44	33	83
GSE50948	9	30	6	11	15	41
Total	39	56	81	132	145	439

Supplementary Notes

Supplementary Note 1.

Results of pathway analysis methods benchmarking according to recently proposed pipeline by Tarca et al[1] are provided in this section. As iPANDA does not produce p-values suitable for pathway relevance assessment, only benchmarking in respect to prioritization criteria from the pipeline was considered. For each dataset a pathway of interest from KEGG database was associated with the disease represented in the dataset, e.g. in a dataset with renal cell tumors, renal cell carcinoma KEGG pathway was expected to be perturbed. Gene content and pathway topology for each pathway were fetched from KEGG database with KEGGgraph[7] and KEGG.db (http://bioconductor.org/packages/release/data/annotation/html/KEGG.db.html) R packages. We considered only those pathways for which we could successfully fetch pathway topology with KEGGgraph parseKGML2Graph function, which limited the analysis to 229 KEGG pathways. This list included all pathways of interest from the benchmarking pipeline[1]. As previously described[1], we have performed calculations with each of the analysis methods under study (iPANDA, PADOG[2], ORA[3], PLAGE[4], ssGSEA[5] and two versions of MIPA[6]) on each of the datasets from the pipeline. iPANDA was run with default parameters. iPANDA scores for each pathway were normalized by the division on the sum of topological weights within given pathway to make scores for different pathways comparable. Pathway ranks were defined as inverse ranks of absolute mean values of normalized iPANDA pathway activation scores in all affected samples (more perturbed pathways had lower ranks). PADOG and ORA were executed with the script provided by Adi Tarca[1] with the number of permutations set to 2000. PLAGE and ssGSEA were executed as described in the original manual for GSVA[8] R package: after PLAGE/ssGSEA step, differential pathway activation p-values were calculated with lmFit/eBayes (as described in the tutorial), the ranks of pathways were defined as the ranks of the respective p-values. The results of MIPA on the datasets from the pipeline were kindly provided by the author of MIPA method[6] Gautam Goel. Each method yielded an ordered list of pathways, starting from the most perturbed to the least perturbed. Relative ranks (100 * rank of the pathway / total number of pathways analyzed, where a lower rank indicates more significant perturbation of the pathway) were calculated for the pathway of interest in each of the datasets. Distributions of relative ranks for pathways of interest are represented as boxplots (**Supplementary Fig. 8**). Lower ranks mean higher scores for pathways of interest related to the scores for other pathways.

iPANDA method gives meaningful results, particularly, the pathways expected to be perturbed have significantly lower ranks (higher scores) than if it was expected by chance. The comparison shows that PADOG and both versions of MIPA appeared to perform better than iPANDA, ORA had similar performance, PLAGE and ssGSEA methods had lower performance.

Supplementary Note 2.

To further evaluate the proficiency of iPANDA for reliably classifying a sample with respect to the various clinical parameters, we have used the training and validation datasets (**Supplementary Fig. 9**) along with the best predictive models reported by the MAQC-II project as a reference for comparison. For each oncology MAQC-II dataset used in this study, we have carefully selected a tissue specific normal control (microarrays derived from the healthy subjects) (**Supplementary Table 1**), which were normalized with respect to the original datasets used for prediction of 10 endpoint indicative of oncological conditions by MAQC-II consortium [9]. We next calculated the iPANDA scores for each dataset by using the SA Biosciences signaling pathways knowledge base. All calculations (normalization and generation of iPANDA scores) have been performed independently for training and

validation datasets. Using the iPANDA scores from the training sets, random forest-based gene-level classifier was trained for each oncology endpoint, and its efficacy has been tested using the validation datasets. To demonstrate effectiveness of the iPANDA scores for endpoint prediction, similar classifiers have been trained using four different gene expression subsets: expression levels of all genes, fold change for all genes between the training set and paired normals, expression levels of most differentially expressing genes ($p<0.05$), and fold change in expression levels of most differentially expressing genes ($p<0.05$) between the training and normal datasets). These results have been compared with the data generated by teams participating in the original MAQC-II experiment (overall best team and the best result for each of endpoint analyzed). Moreover, for endpoints D, E, F, G, H and I, we have compared our data with results generated with the best predictors from the IMPROVER DSC challenge [10]. Off note, we were unable to use the IMPROVER code for neuroblastoma datasets used by the MAQC-II project, since these data was generated with Agilent profiling platform, whereas IMPROVER methods were designed to be used with Affymetrix.

Our random forest-based gene-level classifier demonstrates better prediction performance comparing to the average result of MAQC-II teams for majority of the endpoints. Notably, it outperforms MAQC-II candidate gene-level performance models for endpoints J and L. These results indicate the overall enhancement of machine-learning algorithms, since the MAQC experiment was performed seven years ago, and suggest that our particular random-forest implementation can be utilized for further experiments.

The same random forest classifier trained on fold changes (FC) between samples under study and case samples, rather than on pure case samples gene expression, generates the same, or even better results for several 'hard-to-predict' cancer endpoints (D, J and K). This observation demonstrates that incorporating expression data from corresponding normal tissue into the FC calculation, can be valuable addition when developing prediction models. In contrast, using the differentially expressed genes with fixed cutoff, does not result in significant improvement in model prediction performance.

Furthermore, using the iPANDA scores for cancer-related pathways to train prediction models, allows to obtain significantly better results compared to the gene-level prediction models developed by either our team or MAQC-II consortium (for threes out of five cancer endpoints available for comparison F, J, K). The highest increase in performance was achieved on Neuroblastoma event free survival (endpoint K) (0.894 against 0.575 for the best MAQC-II team). These observations further support our notion that iPANDA algorithm can provide an efficient noise reduction when extracting biologically relevant features from the data. Hence iPANDA scores can be used as input for machine learning algorithms including deep neural networks to make better prediction models.

Interestingly, while negative control endpoints (I and M) remained unpredictable for all our models, the MAQC-II positive control gender endpoints (H and L) have also appeared to be unpredictable when using models based on iPANDA scores for cancer-related pathways. These may be attributed to fact that gender-specific genes are poorly represented in cancer-related pathways utilized for prediction. Subsequently, our data indicates that performance of prediction models based on pathway-level data, greatly depends on the pathway database used.

Supplementary References

1. Tarca, A. L., Bhatti, G. & Romero, R. A comparison of gene set analysis methods in terms of sensitivity, prioritization and specificity. *PLoS One* **8,** e79217 (2013).
2. Tarca, A. L., Draghici, S., Bhatti, G. & Romero, R. Down-weighting overlapping genes improves gene set analysis. *BMC Bioinformatics* **13,** 136 (2012).
3. Tavazoie, S., Hughes, J. D., Campbell, M. J., Cho, R. J. & Church, G. M. Systematic

determination of genetic network architecture. *Nat. Genet.* **22,** 281–285 (1999).
4. Tomfohr, J., Lu, J. & Kepler, T. B. 10.1186/1471-2105-6-225. *BMC Bioinformatics* **6,** 225 (2005).
5. Barbie, D. A. *et al.* Systematic RNA interference reveals that oncogenic KRAS-driven cancers require TBK1. *Nature* **462,** 108–112 (2009).
6. Goel, G., Conway, K. L., Jaeger, M., Netea, M. G. & Xavier, R. J. Multivariate inference of pathway activity in host immunity and response to therapeutics. *Nucleic Acids Res.* **42,** 10288–10306 (2014).
7. Zhang, J. D. & Wiemann, S. KEGGgraph: a graph approach to KEGG PATHWAY in R and bioconductor. *Bioinformatics* **25,** 1470–1471 (2009).
8. Hänzelmann, S., Castelo, R. & Guinney, J. GSVA: gene set variation analysis for microarray and RNA-seq data. *BMC Bioinformatics* **14,** 7 (2013).
9. Shi, L. *et al.* The MicroArray Quality Control (MAQC)-II study of common practices for the development and validation of microarray-based predictive models. *Nat. Biotechnol.* **28,** 827–838 (2010).
10. Tarca, A. L. *et al.* Strengths and limitations of microarray-based phenotype prediction: lessons learned from the IMPROVER Diagnostic Signature Challenge. *Bioinformatics* **29,** 2892–2899 (2013).
